# Highly modulated supported triazolium-based ionic liquids: direct control of the electronic environment on Cu nanoparticles†

**DOI:** 10.1039/d0na00055h

**Published:** 2020-02-12

**Authors:** Cristián Valdebenito, Jose Pinto, Michael Nazarkovsky, Gustavo Chacón, Oriol Martínez-Ferraté, Kerry Wrighton-Araneda, Diego Cortés-Arriagada, María Belén Camarada, Jesum Alves Fernandes, Gabriel Abarca

**Affiliations:** Centro de Nanotecnología Aplicada, Facultad de Ciencias, Universidad Mayor Camino la Pirámide 5750 Huechuraba Santiago Chile gabriel.abarca@umayor.cl; School of Chemistry, University of Nottingham NG7 2RD Nottingham UK jesum.alvesfernandes@nottingham.ac.uk; Departamento de Química, Pontifícia Universidade Católica do Rio de Janeiro R. Marquês de São Vicente 225 Rio de Janeiro 22451-900 RJ Brazil; Instituto de Química, Universidade Federal do Rio Grande do Sul Porto Alegre Rio Grande do Sul Brazil; Programa Institucional de Fomento a la Investigación, Universidad Tecnológica Metropolitana Desarrollo e Innovación Ignacio Valdivieso 2409, P.O. Box San Joaquín Santiago Chile

## Abstract

A series of new triazolium-based supported ionic liquids (SILPs), decorated with Cu NPs, were successfully prepared and applied to the N-arylation of aryl halides with anilines. The triazoles moieties were functionalised using copper-catalysed azide–alkyne cycloaddition. SILP surface characterisation showed a strong correlation between the triazolium cation volume and textural properties. STEM images showed well-dispersed Cu NPs on SILPs with a mean diameter varying from 3.6 to 4.6 nm depending on the triazolium cation used. Besides, XPS results suggest that the Cu(0)/Cu(i) ratio can be modulated by the electronic density of triazolium substituents. XPS and computational analysis gave mechanistic insights into the Cu NP stabilisation pathways, where the presence of electron-rich groups attached to a triazolium ring plays a critical role in leading to a cation adsorption pathway (*E*_ads_ = 72 kcal mol^−1^). In contrast, less electron-rich groups favour the anion adsorption pathway (*E*_ads_ = 63 kcal mol^−1^). The Cu@SILP composite with electron-rich groups showed the highest activity for the C–N Ullmann coupling reaction, which suggests that electron-rich groups might act as an electron-like reservoir to facilitate oxidative addition for N-arylation. This strategy firmly suggests the strong dependence of the nature of triazolium-based SILPs on the Cu NP surface active sites, which may provide a new environment to confine and stabilise MNPs for catalytic applications.

## Abstract 2

A series of new triazolium-based supported ionic liquids (SILPs), decorated with Cu NPs, were successfully prepared and applied to the *N*-arylation of aryl halides with anilines.

## Graphical abstract

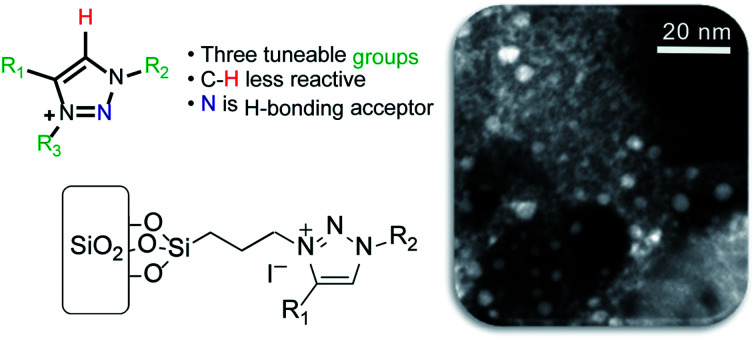

## Introduction

**Fig. 1:**
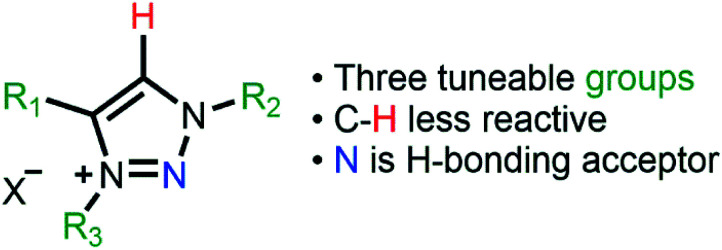
Different functionalities of 1,2,3-triazolium rings as ionic liquids.

Supported metal nanoparticle (MNP) systems have been extensively applied in catalytic processes, in which the metal–support interaction may change the performance, selectivity, and lifetime of the metal catalyst.^1,2^ In general, the interactions between catalysts, substrates, and products are governed by the chemical environment, where the stabilisation of the MNPs plays a critical role in the outcome of the reaction.^3–5^ In this regard, the functionalisation of mesoporous supports (*e.g.*, SiO_2_ and Al_2_O_3_) with suitable ionic liquids (ILs) arose as a promising alternative, which combines textural properties and a tuneable chemical environment.^6^ In this process, a thin IL layer is grafted over mesoporous matrices which controls the textural properties and also creates a confined space for structural and electronic stabilisation of the MNPs.^7^ ILs are described as “entropic drivers” since the cation/anion functionalities can be tuned by the proper selection of each ionic moiety.^8^ For instance, the use of hydrophobic IL anions can control the MNP size and distance from the support matrix and thus modify their catalytic selectivity.^9,10^ Furthermore, acidic SILPs combined with MNPs can act as multifunctional catalysts for the selective hydrodeoxygenation of aromatic substrates.^11^ Despite this exciting research using imidazolium-based SILPs, there is no or very little research using different IL-based SILPs, and a fresh approach can unlock a new generation of SILPs for catalytic applications.^12–14^ In this regard, the highly modulated and greener synthesis of a SILP based-triazolium family appears as an exciting novel alternative. In the case of triazoles, the incorporation of different functional groups into the triazolium ring can be easily obtained using copper-catalysed azide–alkyne cycloaddition (CuAAC), which has emerged as a central transformation of click chemistry (Fig. 1).^15,16^

Moreover, the triazoles moieties are obtained with high regioselectivity and yields (>90%) with no generation of harmful by-products, thus being considered an environmentally friendly approach. Here, we present the synthesis of novel triazolium-based SILPs decorated with Cu NPs for the N-arylation of amines with aryl halides.^17^ The morphological properties of the SILPs and Cu@SILP composites were extensively investigated by Scanning Electron Microscopy (SEM), Scanning Transmission Electron Microscopy (STEM), X-ray Photoelectron Spectroscopy (XPS) and Brunauer–Emmett–Teller (BET) measurements using the Nguyen-Do method^18,19^ modified by Gun'ko (MND).^20,21^ Furthermore, detailed quantum computational studies were carried out, revealing the IL and Cu NP interaction pathway. The catalytic properties of Cu@SILP composites were evaluated by C–N Ullmann condensation of aryl halides with aniline.^22,23^

## Experimental

### Materials

Aniline, iodobenzene, bromobenzene, chlorobenzene, 3-iodopropyltrimethoxysilane, benzyl bromide, phenylacetylene, NaN_3_, CuCl_2_·2H_2_O, CuI, NaBH_4_, MgSO_4_, and BMI·BF_4_ were purchased from Sigma-Aldrich. All chemicals were used without further purification, except for DCM, toluene, MeOH, acetonitrile, Et_2_O, and EtOAc, which were purified by standard procedures.^24^

### Methods

^1^H NMR measurements of the ILs were performed on a Varian 400 MHz. ^29^Si and ^13^C cross-polarisation magic angle spinning (CP-MAS) solid-state measurements of the supports were carried out using a Varian 500 MHz spectrometer. The CP-MAS spectra were recorded using a 2-channel 4 mm Narrow Bore probe and zirconium rotors. Fourier-transform infrared spectra (FT-IR) were obtained on a Bruker Alpha-P spectrometer with a resolution of 4 cm^−1^. Thermogravimetric analyses (TGA) were performed using a Q50 TA TGA operating under a nitrogen gas atmosphere (flux of 40 mL min^−1^). The specific surface area was obtained from the nitrogen physisorption isotherms using the BET surface area model and MND (see the ESI† for details). SEM analyses were carried out using a Zeiss Auriga (FE-SEM) at 30 kV. The STEM analyses were performed with an XFEG Cs corrected FEI Titan 80/300 microscope. High Z-contrast images were acquired through STEM using a high-angle annular dark-field detector (HAADF). Powder X-ray Diffraction (XRD) spectra were recorded using a voltage of 30 kV and current at 25 mA within range of 2*θ* = 20–90° (Cu-K_α_ = 0.154 nm). Inductively Coupled Plasma Optical Emission Spectroscopy (ICP-OES) measurements were performed using a PerkinElmer Optima 2000 DV ICP-OES at a selected wavelength of Cu (374.747 nm). XPS measurements were carried out using a Kratos AXIS Ultra DLD instrument. The chamber pressure during the measurements was 5 × 10^−9^ Torr. Wide energy range survey scans were collected at a pass energy of 80 eV in hybrid slot lens mode and a step size of 0.5 eV. High-resolution XPS data on the C 1s, O 1s, N 1s, I 3d, and Cu 2p photoelectron peaks were collected at a pass energy of 20 eV over energy ranges suitable for each peak, with a collection time of 5 min and a step size of 0.1 eV. The X-ray source was a monochromated Al K_α_ emission operated at 10 mA and 12 kV (120 W). The energy range for each pass energy (resolution) was calibrated using the Kratos Cu 2p_3/2_, Ag 3d_5/2_, and Au 4f_7/2_ three-point calibration method. The data were charge corrected to the reference carbon adventitious signal at 284.8 eV.

### Synthesis of triazole1–3

Alkyne (5 mmol, 1.0 eq.), alkyl halide (1.0 eq.), and NaN_3_ (1.3 eq.) were loaded into a 25 mL round-bottom flask. Then [CuI(PPh_3_)_3_] (0.05 mol%) was added and dissolved in water (5 mL). The reaction was stirred for 4 h at room temperature, and the progress of the reaction was monitored using thin-layer chromatography (TLC). After its completion, the reaction mixture was filtered, and the residue was dissolved in DCM. The combined organic layer was later concentrated in a vacuum to yield the corresponding triazoles. The products were characterised by spectroscopic analysis as described before.

### Synthesis of SiO_2_–I

Initially, 200 g mesh silica gel was soaked in 30% HCl overnight to hydrolyse its surface. Next, activated silica gel (100 g) was suspended in dry toluene (30 mL) in a round-bottom flask equipped with a reflux condenser under nitrogen. While being stirred, 3-iodopropyltrimethoxysilane (0.05 M) was added dropwise. The suspension was refluxed for 72 h. After cooling, the solid was collected by filtration and exhaustively washed by Soxhlet extraction with ethanol and water, and then dried under reduced pressure to yield iodopropyl–silica gel (SiO_2_–I).^25^

### Synthesis of SILP1–3

**Scheme 1:**
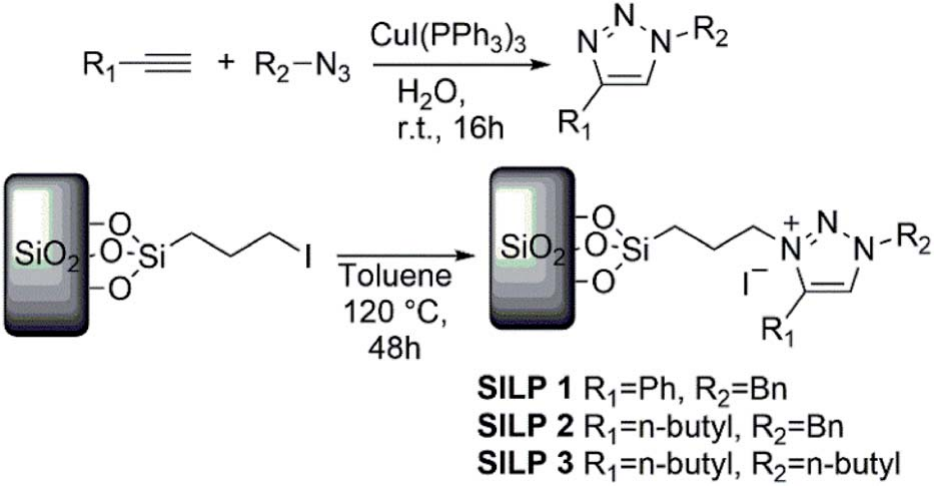
Synthetic route of SILP materials.

SiO_2_–I (5 g) was suspended in dry acetonitrile (30 mL), with stoichiometric amounts of triazoles1–3 (see Scheme 1 for details) and refluxed during 24 h. Then, the particles were collected by filtration and repeatedly washed with Et_2_O and water, and then dried off under vacuum at 110 °C for 4 h to obtain SILP1–3 powder.

### Synthesis of Cu@SILP1–3

A solution of CuCl_2_·2H_2_O (0.25 mmol) and MeOH (20 mL) was added to SILP1–3 (100 mg) under constant stirring at room temperature for 30 min. A solution of NaBH_4_ (5 mmol) dissolved in MeOH (3 mL) was added to the reaction mixture dropwise. The reaction mixture turned black due to the formation of Cu NPs that were washed with MeOH (3 × 10 mL) and Et_2_O (3 × 10 mL). Subsequently, the samples were isolated by centrifugation (4500 rpm) and dried under reduced pressure.^26^ Neat Cu_2_O@SiO_2_ was synthesised employing the same procedure reported above using CuI as a precursor.

### Computational section

DFT calculations were performed using the ORCA 4.1 program^27^ with the Perdew–Burke–Ernzerhof (PBE)^28^ functional. All-electron 6-31G(d) basis sets were used for H, C, N, O, and Si atoms;^29,30^ the LANL2DZ pseudopotential and basis set was employed for Cu and I atoms.^31^ The def2/J auxiliary basis set was implemented for the resolution-of-identity procedure in the ORCA program.^32^ The DFT method was previously validated (see the ESI† for details). Basis-set superposition errors were corrected using the geometrical counterpoise method.^33^ Dispersion energy corrections were included through the atom-pairwise DFT-D3 methodology with the Becke–Johnson damping function.^34^ The Cu_55_ cluster model was selected^35^ to represent the Cu NPs, while the SILP structures were truncated at the Si–O link. A conformational search by semiempirical molecular dynamics (MD) was performed using the Gabedit 2.5 program^36^ to obtain the minimum energy structures of the SILPs. The most stable conformations were used as input structures for re-optimisation by DFT calculations. The potential in MD calculations was determined “on-the-fly” at the PM6 level in MOPAC2016,^37^ and the atomic positions were updated by using the Verlet velocity algorithm.^38^ The stability of the SILPs and Cu_55_ was evaluated using the adsorption energy (*E*_ads_) as follows:

1 *E*_ads_ = *E*_Cu_ + *E*_ion_ − *E*_Cu-ion_

where *E*_Cu_ is the energy of the Cu_55_ cluster, *E*_ion_ is the energy of the isolated ion, and *E*_Cu-ion_ is the energy of the Cu_55_-ion cluster. The more positive and stable value of *E*_ads_ leads to the formation of composites and therefore higher strength to the interaction between SILP and Cu_55_. The charge transfer was evaluated using the fragmental charge (*q* as the sum of atomic charges in one fragment) by Löwdin population analysis.^39^ Consequently, the charge transfer of fragment i (Δ*q*_i_) is defined as the difference between fragmental charge before (*q*_ib_) and after (*q*_ia_) the interaction.

### N-Arylation of aryl halides with aniline

In a typical reaction, amine (1.2 mmol), aryl halide (1 mmol), base (2 mmol), Cu@SILP1–3 (1 mol%), and solvent (3 mL) were added to a round-bottom flask. The reaction mixture was stirred at 120 °C for 4 h. After the completion of the reaction, the mixture was cooled and extracted with water/EtOAc. The combined organic extracts were dried with anhydrous MgSO_4_, and the solvent was removed by rotary evaporation. The conversion and selectivity were determined by GC analysis.

## Results and discussion

### Preparation and characterisation of SILP1–3

**Fig. 2:**
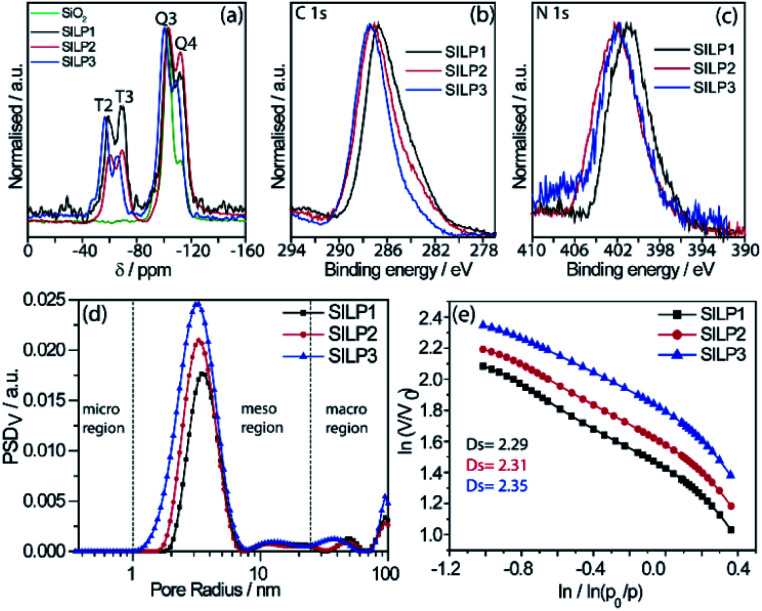
(a) ^29^Si CP-MAS spectrum obtained for SILP1–3, (b) high-resolution XPS spectra of SILP1–3 for (b) C 1s and (c) N 1s regions, (d) PSD profiles in volume (PSDv) of SILP1–3, and (e) Frenkel–Halsey–Hill plot for SILP1–3.

The SILPs were prepared by covalent attachment of triazolium moieties on a silica matrix. The 1,4-disubstituted 1,2,3-triazole moieties were prepared with high regioselectivity and yields (>90%) by click chemistry of an alkyne and *in situ* generated azide. The prepared triazole was further alkylated to afford 1,3,4-trisubstituted 1,2,3-triazolium compounds (Scheme 1, see the ESI† for details).^40^ In this work, we prepared supports with high electronic density (SILP1) by the addition of phenyl and benzyl groups to the triazolium ring and supports with lower electronic density (SILP2–3) replacing the aromatic groups by an alkyl chain. Solid-state ^29^Si NMR and CP-MAS ^13^C NMR analyses confirmed the presence of silanol groups and the incorporation of alkyl silanes on the silica surface (Fig. 2a and S1†). The chemical environments of Si atoms are associated with unreacted Si–C bonds and the T^2^ and T^3^ species that confirm the presence of unchanged triazolium ILs in SILP1–3 (Tables S1 and S2†).^41^ FT-IR spectra show two characteristic peaks, at 1458 cm^−1^ and 1570 cm^−1^, associated with the stretching frequencies of the triazolium ring,^42^ and a sharp peak at 1190 cm^−1^ related to *ν*_as_(Si–O–Si)_L,T_, which confirms the silica matrix functionalisation (Fig. S2†).^41^ TGA measurements were carried out to verify and quantify the incorporation of alkyl iodosilane and triazolium moieties over the silica surface and to determine their thermal properties. SILP1–3 showed similar weight loss behaviour with increasing temperature. The first weight loss region (*ca.* 100 °C) is associated with adsorbed water confined into the porous silica matrix, whereas the second weight loss observed at 200–400 °C is ascribed to IL moieties (SILP1–3).^43^ The loading of ILs into the silica matrix was found to be *ca.* 25–28%, as expected (Fig. S3†). The chemical surface composition of the SILPs was evaluated by XPS (Fig. S4†). The ratio of N 1s to Si 2p corresponds approximately to the expected ratio between triazolium and the silica matrix (Table S3,† entries 1–3). The C 1s and N 1s signals displayed a positive shift for SILP1 against SILP2–3 (Fig. 1b and c), attributed to the electronic effect promoted by the aromatic groups (SILP1), in comparison to ILs with alkyl chains at the triazolium ring (SILP2–3).^44^

The textural properties of the SILPs were evaluated by BET measurements and analysed by MND that suggests the presence of porous-like cylinders for all SILPs. From BET measurements, the specific surface area of SILP1, SILP2, and SILP3 increases congruently with the total pore volume and average pore radius moving towards the nanopore region (Fig. 2d). The MND analysis shows similar values of *S*_meso_/*S*_BET_ (higher than 99%) and an increase of the *S*_meso_ absolute value in the order SILP1 < SILP2 < SILP3 (Table S4†). Also, PSD_V_ (pore size distribution by volume) and PSD_S_ (pore size distribution by specific surface area) show a shift to smaller radii in SILP1 < SILP2 < SILP3 within the mesoporous range (Table S5†). These results are in good agreement with Nonlocal density functional theory (NLDFT) and Barrett–Joyner–Halenda (BJH) analyses (Fig. S5, Table S5†).^45^ Additionally, the surface roughness degree and porous structure of the SILPs were determined by fractal dimension (*D*_s_) analysis employing the Frenkel–Halsey–Hill plot, where for a “flat” surface, *D*_s_ = 2, whereas for an irregular (real) surface, *D*_s_ varies between 2 and 3.^46^ The apparent decreases of roughness SILP1 < SILP2 < SILP3 (Fig. 2e) follows the same trend as the specific surface area and PSD_V_–PSD_S_ analysis (Fig. 2d, Tables S4 and S5†).^47^ These results suggest that different substituents over the triazolium ring can directly modify the textural properties of the silica matrix. The addition of large substitutes, such as SILP1 (*e.g.*, phenyl-benzyl groups), led to a decrease in the surface area, roughness, and pore volume when compared to SILP2–3. This behavior coincides with observed nitrogen adsorption energy distribution which follows the trend SILP1 < SILP2 < SILP3 (Fig. S6†). FE-SEM showed substantial changes for silica surface roughness after the incorporation of the triazolium-based ILs (Fig. S7†). In addition, the FE-SEM images suggest that the SILP1–3 surface exhibited wavy and rope-like mesoporous domains corroborating the MND analysis.

### Characterisation of Cu@SILP1–3

**Fig. 3:**
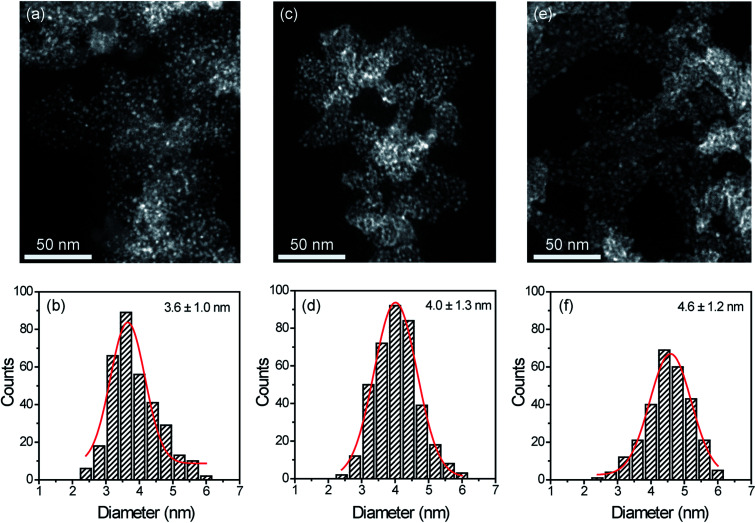
STEM images and histograms of (a and b) Cu@SILP1, (c and d) Cu@SILP2 and (e and f) Cu@SILP3.

**Fig. 4:**
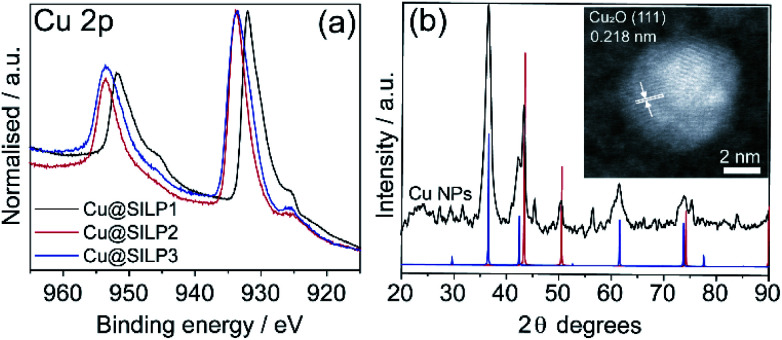
(a) High-resolution XPS for the Cu 2p region; (b) XRD of isolated Cu NPs (black line) and XRD patterns of Cu (#53247 — red line) and Cu_2_O (#52043 — blue line); and inset shows STEM of Cu NP (111) lattice fringes of 0.218 nm.

**Fig. 5:**
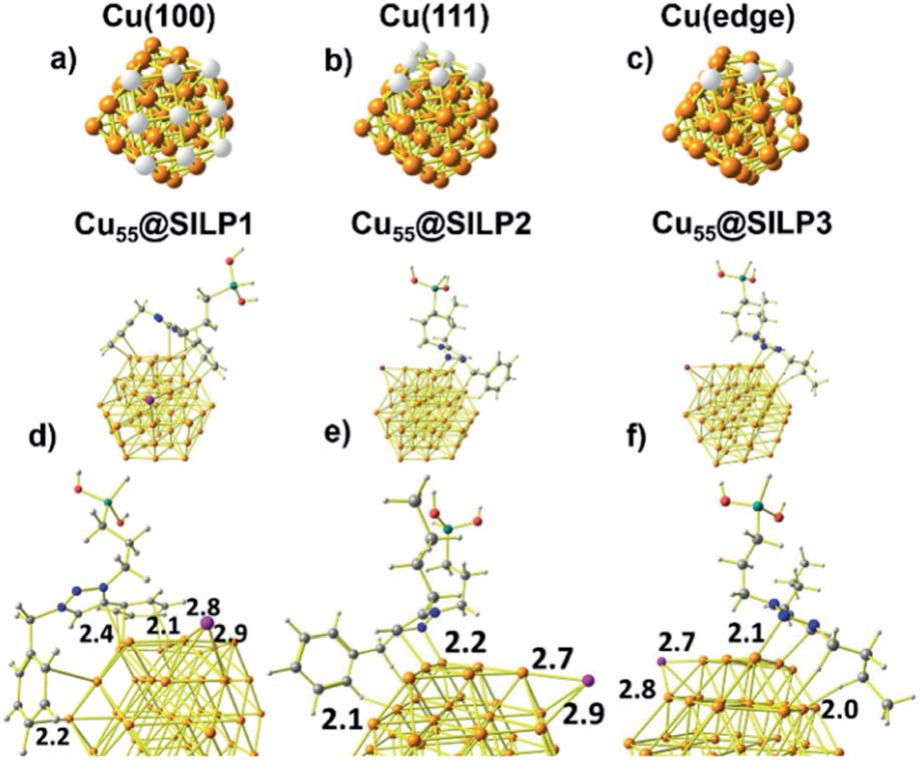
(a) Cu(100) face, (b) Cu(111) face, and (c) Cu(edge) atoms of the Cu_55_ cluster. The respective Cu atoms of the facet are highlighted in white. Minimum energy structures of the (d) Cu_55_@SILP1, (e) Cu_55_@SILP2 and (f) Cu_55_@SILP3 complexes. Distances are in Angstrom (Å).

STEM measurements showed that Cu NPs were homogeneously dispersed on the surface of SILPs displaying a mean diameter of 3.6 ± 1.0 nm, 4.0 ± 1.3 nm, and 4.6 ± 1.2 nm for Cu@SILP1, Cu@SILP2, and Cu@SILP3, respectively (Fig. 3, S8, S9 and S10†). It can be related to lower roughness and porous size (SILP1 < SILP2 < SILP3), as smaller pores can act as cavities for the stabilisation of smaller NPs. Further insights into Cu NP electronic stabilisation by the ILs were obtained by XPS (Fig. 4a and S11†), XRD (Fig. 4b), and computation analysis (Fig. 5). XPS measurements of Cu@SILP1–3 showed a broad peak at 932.2 eV (Cu 2p_3/2_) and 951.9 eV (Cu 2p_1/2_) that can be assigned to a mixture of valence states Cu(0) and Cu(i).^48,49^ The XRD analysis also confirms the presence of metallic Cu and Cu_2_O, in agreement with XPS results, as well as STEM analysis that shows Cu NP lattice fringes (111) associated with the FCC crystalline phase of Cu_2_O (Fig. 4b – inset).^50^ In order to qualitatively estimate the Cu(0) and Cu(i) ratio, Cu LMM analysis was performed indicating a majority presence of Cu(i) valence states for Cu@SILP1–3 (Fig. S12†). We ascribed the stabilisation of Cu(i) to the confined environment provided by IL pairs to the copper surface. Additionally, a shift towards higher kinetic energy in Cu LMM spectra for Cu@SILP1, as well as a shift towards lower binding energy in the Cu 2p spectra, compared to Cu@SILP2–3 was observed.^51^ Interestingly, a similar shift of cations (C 1s and N 1s) for SILP1 compared to SILP2–3 was detected before the Cu NP deposition (Fig. 1b, c, and 4a), which indicates a strong effect of the IL cation on the Cu NP electronic environment. Furthermore, a small shoulder at 932.5 eV was associated with iodide ion and Cu NP interactions.^48^ In summary, these results demonstrate that both cations and anions of the IL play have an effect on Cu NP valence states, which is further investigated by computational studies.

### Computational analysis of the SILPs–CuNP interaction

Computational studies were performed to understand the stabilisation/interaction between SILP1–3 and Cu NPs. Cu_55_ clusters with six Cu(100) and eight Cu(111) faces were chosen in order to represent the Cu NPs synthesised in this work (Fig. 5a and b). Each Cu(100) and Cu(111) pair of faces shares three Cu atoms at the edges [Cu(edge) atoms] (Fig. 5c). Cu_55_ shows Cu–Cu bond lengths in the range of 2.4–2.5 Å in agreement with crystallographic data for metallic Cu (2.55 Å).^52^

With the computational method, we first examined the interactions presents on Cu_55_@SILP1 (Fig. 5d). The triazolium ring interacts with Cu(111) and Cu(edge) atoms at a 2.4 Å bond length, whereas phenyl and benzyl show intermolecular bond lengths of 2.1 and 2.2 Å on Cu(100) and Cu(edge) atoms, respectively. In Cu_55_@SILP2, the cation interacts through the benzyl hydrogen and the triazole group with Cu(edge) atoms at bond lengths of 2.1 to 2.2 Å, respectively (Fig. 5e). In the case of Cu_55_@SILP3, the triazole ring interacts with Cu(edge) atoms at a bond length of 2.1 Å, and the butyl groups of the triazolium interact with Cu_55_ at a hydrogen bond length of 2.0 Å (Fig. 5f). The iodide anion, SILP1–3, interacts with Cu atoms with bond lengths of up to ∼2.9 Å. Note that for Cu@SILP2–3 the triazolium cation tends to interact with two faces of Cu_55_, whereas the triazolium cation of SILP1 interacts with at least three faces of Cu_55_. These results demonstrate that SILP1 covers a larger surface of Cu_55_ compared with SILP2–3, thus providing a high contact between the IL (SILP1) and Cu atoms.

**Fig. 6:**
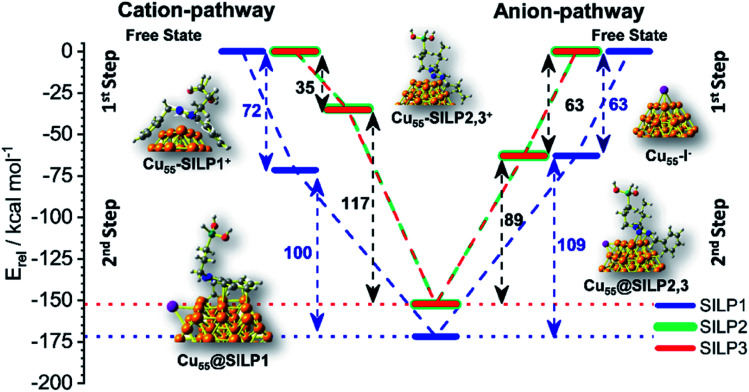
Energy diagram (in relative energy, *E*_rel_) of the co-adsorption of SILPs onto Cu_55_ from the free state of the fragments until Cu_55_@SILP1–3 pass through the first cation adsorption pathway or first anion adsorption pathway.

The adsorption energy (*E*_ads_) was calculated to evaluate the strength of the interaction between SILP1–3 and Cu_55_. A competitive model based on co-adsorption of both IL cations and anions on the Cu_55_ surface was employed considering the free states of each fragment as a starting point (Fig. 6).^53^ For Cu_55_@SILP1, the cation pathway was found to be preferred due to its higher *E*_ads_ (72 kcal mol^−1^) compared to the anion pathway (*E*_ads_ = 63 kcal mol^−1^). The subsequent addition of iodide anions to the system gives an overall *E*_ads_ of 172 kcal mol^−1^. For Cu_55_@SILP2–3, the anion pathway is more favoured as the *E*_ads_ is higher (*E*_ads_ = 63 kcal mol^−1^) compared with the cation pathway (*E*_ads_ = 35 kcal mol^−1^).

The complete *E*_ads_ for Cu_55_@SILP2–3 including their respective cations is 152 kcal mol^−1^. The distinct pathways of IL adsorption on the Cu_55_ surface of SILP1 compared with SILP2–3 are found to be related to the minimum energy arrangement of the IL cations on the Cu_55_ surface (Fig. 5). As discussed previously, SILP1 has two electron-rich groups that interact with three faces of the Cu_55_ cluster, thus favouring the cation pathway, whereas the relatively weaker interaction of SILP2–3 cations with Cu_55_ surfaces leads to a more favourable anion pathway. The total comparative *E*_ads_ of Cu_55_@SILP1 (172 kcal mol^−1^) and *E*_ads_ of Cu_55_@SILP2–3 (152 kcal mol^−1^) suggest a weaker interaction of SILP2–3 with the surface of Cu NPs when compared to SILP1. Furthermore, the overall electron transfer between SILP1–3 and Cu_55_ clusters (Δ*q*^Cu_55_@SILP^) was calculated using the Δ*q* descriptor for each fragment (Table 1). The iodide anion acts as an electron-donating fragment, whereas the electron-acceptor fragments are both Cu_55_ and triazolium cations. The Δ*q*^Cu_55_@SILP^ shows a higher charge transfer from iodide anions to both Cu_55_ and the triazolium cations according to the following trend SILP1 > SILP2 ≈ SILP3. The charge transferred from iodide is distributed between triazolium cations and Cu_55_, which can be divided into two fragmental contributions Δ*q*^Cu_55_^ and Δ*q*^triazolium^. The Δ*q*^Cu_55_^ shows values going from 0.19 to 0.49|*e*|, with Cu_55_@SILP1 showing a higher |*e*| transfer value. The same trend was observed for Δ*q*^triazolium^, with SILP1 showing higher |*e*| values than SILP2–3. These results reinforced the major role of the electronic density of triazolium substituents in the stabilisation of Cu NPs.

**Table 1:** Fragmentary electron transfer (Δ*q*)|*e*| between the fragments Cu_55_, IL anion, and cation for Cu_55_@SILP1–3

Samples	Δ*q*^Cu_55_@SILP^	Δ*q*^Cu_55_^	Δ*q*^triazolium^
Cu_55_@SILP1	0.88	0.49	0.39
Cu_55_@SILP2	0.52	0.19	0.34
Cu_55_@SILP3	0.58	0.28	0.30

### Catalytic activity of Cu@SILP1–3

The catalytic activity of the Cu@SILP1–3 composites was evaluated in the C–N Ullmann coupling reaction to generate diphenylamine selectively from aryl halides (iodo, bromo, and chlorobenzene) and aniline. This model reaction was proposed to investigate the IL steric and electronic environment effect on the Cu NP surface during the catalytic cycle. Catalytic experiments using KOH, K_2_CO_3_, Cs_2_CO_3_ and N(Et)_3_ were performed (Table S6†). KOH has shown the highest catalytic conversion compared to other bases used, which we ascribe to its stronger basicity that may enhance the formation of the amido-complex, and thus KOH was used in all reactions.^54^ All SILPs displayed high selectivity towards the N-arylated product, with traces of triphenylamine and biphenyl as side-products (Table 2). The catalytic activity of Cu@SILP1–3 was observed in the following order Cu@SILP1> Cu@SILP2 > Cu@SILP3 (entries 1–3). It is important to mention that no significant difference in the catalytic activity between Cu@SILP1 and Cu@SILP2 was observed for iodobenzene, whereas for bromo- and chloro-derivatives a significant difference was observed (entries 4, 5, 7 and 8). This behaviour can be related to the faster C–X activation for iodo-derivatives compared to bromo- and chloro-derivatives. These results are in agreement with previous reports where the coupling product can be modulated by the reactivity of the aryl halide compound (R–I ≫ R–Br > R–Cl).^55^ Additionally, neat Cu_2_O NPs were tested in the absence of SILPs (Table 2, entry 10). No significant diphenylamine production was observed, which might be due to a weak stabilisation of Cu(i)/Cu(iii) moieties during the catalysis cycle, in the absence of ILs (SILPs).

**Table 2:** Catalytic properties of Cu@SILP1–3 for C–N Ullmann coupling reactions^a^

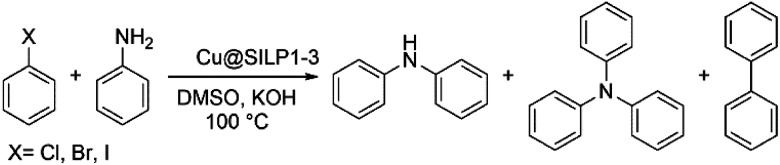					
Entry	Cat	X	Conv (%)	Sel (%)	TOF^b^ (h^−1^)
1	Cu@SILP1	I	88	95	374
2	Cu@SILP2	I	83	94	311
3	Cu@SILP3	I	77	94	301
4	Cu@SILP1	Br	61	92	184
5	Cu@SILP2	Br	48	91	150
6	Cu@SILP3	Br	44	93	164
7	Cu@SILP1	Cl	44	90	132
8	Cu@SILP2	Cl	31	86	103
9	Cu@SILP3	Cl	30	86	92
10	Cu_2_O@SiO_2_^c^	I	35	81	161

a Reaction conditions: Cu@SILP1–3 (1 mol%), aniline (1.2 mmol), aryl halide (1 mmol), KOH (2 mmol), DMSO (3 mL), 120 °C, and 4 h.

b Conversion determined by GC. TOF = mol diphenylamine converted/(mol Cu surface × time). Calculated from the slope of plots of TON *vs. t* at low substrate conversions.^58^

c Neat Cu_2_O NPs supported on SiO_2_ without an ionic liquid (mean diameter 9.5 ± 2.0 nm).

Based on these results, two possible behaviours can be suggested: (i) the presence of an electron-rich IL layer (SILP1 > SILP2 > SILP3) may favour the oxidative addition that involves Cu(i)/Cu(iii) as a rate-determining step (RDS) of this reaction under these conditions.^56^ Therefore, it would be reasonable to conclude that a preferential Cu(i) species over the surface might facilitate the oxidative addition.^54^ (ii) Reduced yields were achieved for different halogen sources, because of the low reactivity observed for deactivated aryl halides on nucleophilic substitutions (C–X: I > Br > Cl).^57^

**Fig. 7:**
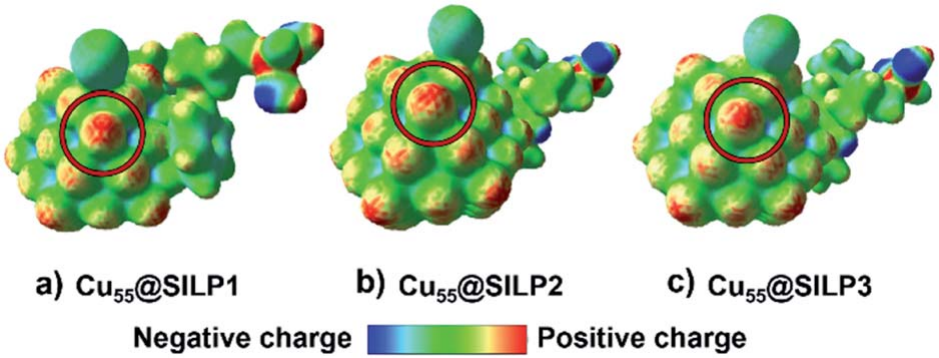
Molecular electrostatic potential (MEP) of the (a) Cu_55_@SILP1, (b) Cu_55_@SILP2 and (c) Cu_55_@SILP3 complexes. The isodensity value is 0.025 a.u. The red and blue colours indicate the zones with charge deficiency and charge excess, respectively. The possible site for the Ullmann catalytic reaction is marked with a red ring.

In this regard, the molecular electrostatic potential (MEP) was calculated for Cu@SILP1–3. The MEP results show that charge polarisation takes place on Cu NPs after the charge transfer between SILPs and Cu NPs (Fig. 7). This process occurs due to a surface charge imbalance between the iodide anion and neighbouring Cu(edge) atoms that present higher positive polarisation.^59^ Therefore, as Cu@SILP1 presented a higher overall electron transfer (between the IL and Cu), it can increase its catalytic activity for Ullmann coupling, considering the oxidative addition step as the RDS.^54,56^ In this regard, we suggest that the IL cation (SILP1) might work like an “electron-like reservoir” that improves the rates of Cu(i)/Cu(iii) redox reactions, and thus, its catalytic activity compared to SILP2–3 (Tables 1 and 2). These results are supported by the Brønsted–Evans–Polanyi (BEP) relationship^59^ that links the MNP stabilisation with catalytic activity. It employs the *E*_ads_ and BEP relationship to explain the catalytic activity of MNPs.^60^ A recent study based on Cu clusters of different sizes (from Cu_13_ to Cu_77_, including Cu_55_ clusters) compared the *E*_ads_ and the catalytic activity in the CO_2_-hydrogenation reaction to methanol showing similar results to this work.^61^

## Conclusions

A new series of triazolium-based SILPs were successfully synthesised and decorated with Cu NPs. The triazolium ring was modified with aromatic and alkyl groups in order to study the influence on the steric and electronic properties of the SILPs. The textural analysis demonstrated that the surface area, porous volume, and roughness decrease according to the volume of substituents in the triazolium cation, thus showing the high tunability of triazolium-based SILPs. Moreover, the stabilisation of Cu NPs seems to be related to the surface properties of the obtained SILPs as the mean diameter of the Cu NPs decreases according to the SILP porous size.

Furthermore, XPS and computational investigations showed that the electronic density of the triazolium cation substituents plays a vital role in the stabilisation pathway of the Cu NPs. In the case of SILP1, two aromatic groups (electron-rich) were incorporated into the triazolium ring leading to a preferential stabilisation of Cu NPs through the cation pathway. In opposition, when alkyl groups are added, the anion pathway is more favourable. The increase of overall *E*_ads_ calculated for Cu@SILPs follows the order Cu@SILP1 > Cu@SILP2 ≈ Cu@SILP3. The computational studies suggest a stronger interaction between SILP1 and Cu NPs occurs due to the higher charge transfer between the IL and Cu atoms. The catalytic results revealed that Cu@SILP1 displayed the best catalytic activity compared to Cu@SILP2–3 for the C–N Ullmann coupling reaction. We suggest that the IL cation of SILP1 may act as an electron-like reservoir adjusting the Cu atom electronic environment (increasing the Cu(i)/Cu(iii) ratio). This behaviour may favour the oxidation state as a RDS, leading to a higher activity compared to Cu@SILP2–3.

Indeed, the triazolium-based SILP approach is easily transferable for other catalytic platforms, and thus this work will open new avenues to design supported metal catalysts for many other catalytic applications.

## Conflicts of interest

The authors declare no conflict of interest.

## Supplementary Material

NA-002-D0NA00055H-s001
