# Correction: Highly modulated supported triazolium-based ionic liquids: direct control of the electronic environment on Cu nanoparticles

**DOI:** 10.1039/d0na90022b

**Published:** 2020-05-12

**Authors:** Cristián Valdebenito, Jose Pinto, Michael Nazarkovsky, Gustavo Chacón, Oriol Martínez-Ferraté, Kerry Wrighton-Araneda, Diego Cortés-Arriagada, María Belén Camarada, Jesum Alves Fernandes, Gabriel Abarca

**Affiliations:** Centro de Nanotecnología Aplicada, Facultad de Ciencias, Universidad Mayor Camino la Pirámide 5750 Huechuraba Santiago Chile gabriel.abarca@umayor.cl; School of Chemistry, University of Nottingham Nottingham NG7 2RD UK jesum.alvesfernandes@nottingham.ac.uk; Departamento de Química, Pontifícia Universidade Católica do Rio de Janeiro R. Marquês de São Vicente 225 Rio de Janeiro 22451-900 RJ Brazil; Instituto de Química, Universidade Federal do Rio Grande do Sul Porto Alegre Rio Grande do Sul Brazil; Programa Institucional de Fomento a la Investigación, Desarrollo e Innovación, Universidad Tecnológica Metropolitana Ignacio Valdivieso, 2409, P. O. Box 8940577 San Joaquín Santiago Chile dcortes@utem.cl

## Abstract

Correction for ‘Highly modulated supported triazolium-based ionic liquids: direct control of the electronic environment on Cu nanoparticles’ by Cristián Valdebenito *et al.*, *Nanoscale Adv.*, 2020, **2**, 1325–1332. DOI: 10.1039/d0na00055h.

The authors regret that the one of the affiliations (affiliation *e*) was incorrectly shown in the original manuscript. The corrected list of affiliations is as shown above.

The Royal Society of Chemistry apologises for these errors and any consequent inconvenience to authors and readers.

## Supplementary Material

