# Supplementary material for: Highly modulated supported triazolium-based ionic liquids: direct control of the electronic environment on Cu nanoparticles
Source: Nanoscale Adv. 2020 Feb 12;2(3):1325–32. doi: 10.1039/d0na00055h (PMC9418861; doi:10.1039/d0na00055h)
Supplement: NA-002-D0NA00055H-s001 [file NA-002-D0NA00055H-s001.pdf]

Electronic Supplementary Material (ESI) for Nanoscale Advances.
This journal is © The Royal Society of Chemistry 2020

Supporting Information

Highly modulated supported triazolium-based ionic liquids: direct control of the electronic environment on Cu nanoparticles

Cristián Valdebenito,[a] Jose Pinto,[b] Michael Nazarkovsky,[c] Gustavo Chacón,[d] Oriol Martínez-Ferraté,[d] Kerry Wrighton-Araneda,[e] Diego Cortés-Arriagada,[e] María Belén Camarada,[a] Jesum Alves Fernandes,*[b] and Gabriel Abarca*[a]

[a]Centro de Nanotecnología Aplicada, Facultad de Ciencias, Universidad Mayor, Camino la Pirámide 5750, Huechuraba, Santiago, Chile
[b]School of Chemistry, University of Nottingham, NG7 2RD, Nottingham, UK.
[c]Departamento de Química, Pontificia Universidade Católica do Rio de Janeiro, Rio de Janeiro, Brazil.
[e]Instituto de Química, Universidade Federal do Rio Grande do Sul, Porto Alegre, Rio Grande do Sul, Brazil.
[d]Programa Institucional de Fomento a la Investigación, Desarrollo e Innovación, Universidad Tecnológica Metropolitana, Ignacio Valdivieso 2409, P.O. Box San Joaquín, Santiago, Chile.

E-mail: jesum.alvesfernandes@nottingham.ac.uk, gabriel.abarca@umayor.cl

1. Support Phase Ionic Liquid (SILP) Characterisation

1.1 ^{13}C CP-MAS NMR

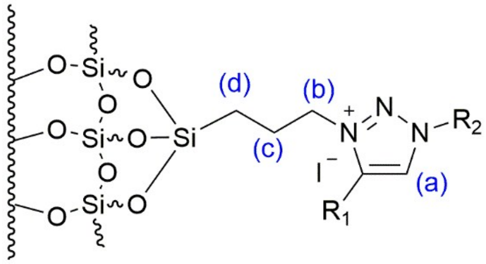

SILP1 R_1=Ph, R_2=Bn
SILP2 R_1=*n*-butyl, R_2=Bn
SILP3 R_1=*n*-butyl, R_2=*n*-butyl

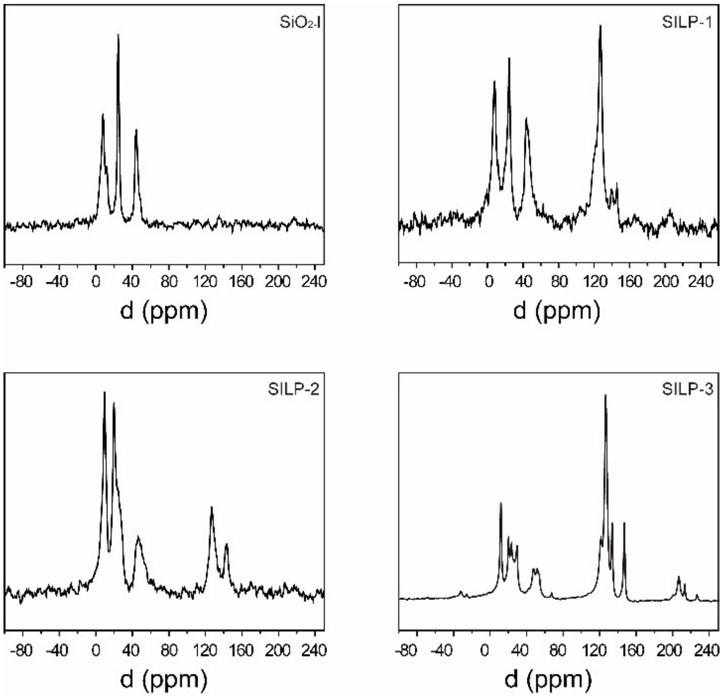

Figure S1. ^{13}C CP-MAS NMR spectres for SILP1-3.

Table S1. ^{13}C CP-MAS NMR data for SILP1-3 supports.

Support	(a)	(b)	(c)	(d)
SiO_2-I	-	44.5	24.1	7.8
SILP1	127.1	43.7	24.8	8.3
SILP2	126.9	46.7	20.9	9.2
SILP3	126.9	48.0	20.9	11.8

Table S2. ^{29}Si CP-MAS NMR data for SILP1-3 supports

Support	T^1	T^2	T^3	Q^1	Q^2	Q^3	Q^4
SiO_2					-92.8	-102.8	-112.2
SILP1		-58.9	-69.3			-103.2	-111.9
SILP2		-60.4	-69.3			-103.7	-112.2
SILP3	-49.7	-59.1	-68.1			-102.9	-111.5

1.2 FT-IR spectra

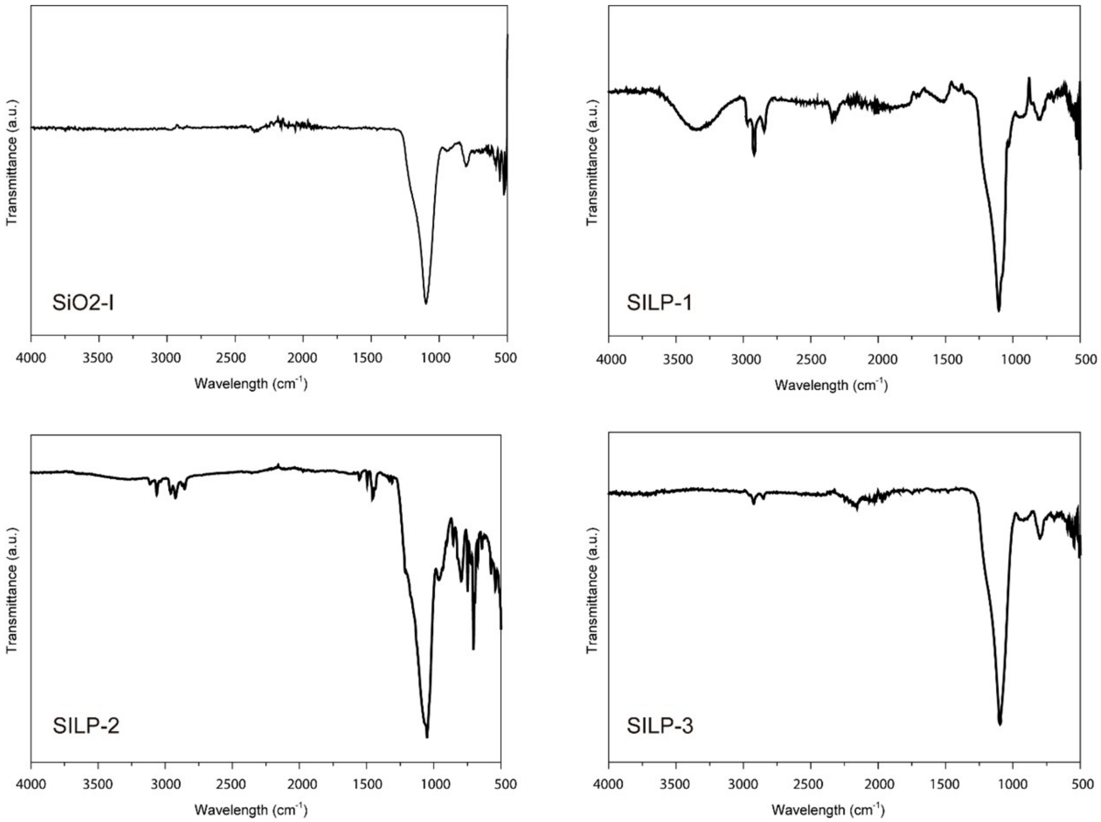

Figure S2. FTIR spectra corresponding to SILP1-3.

1.3 Thermogravimetric analyses - TGA and DTG

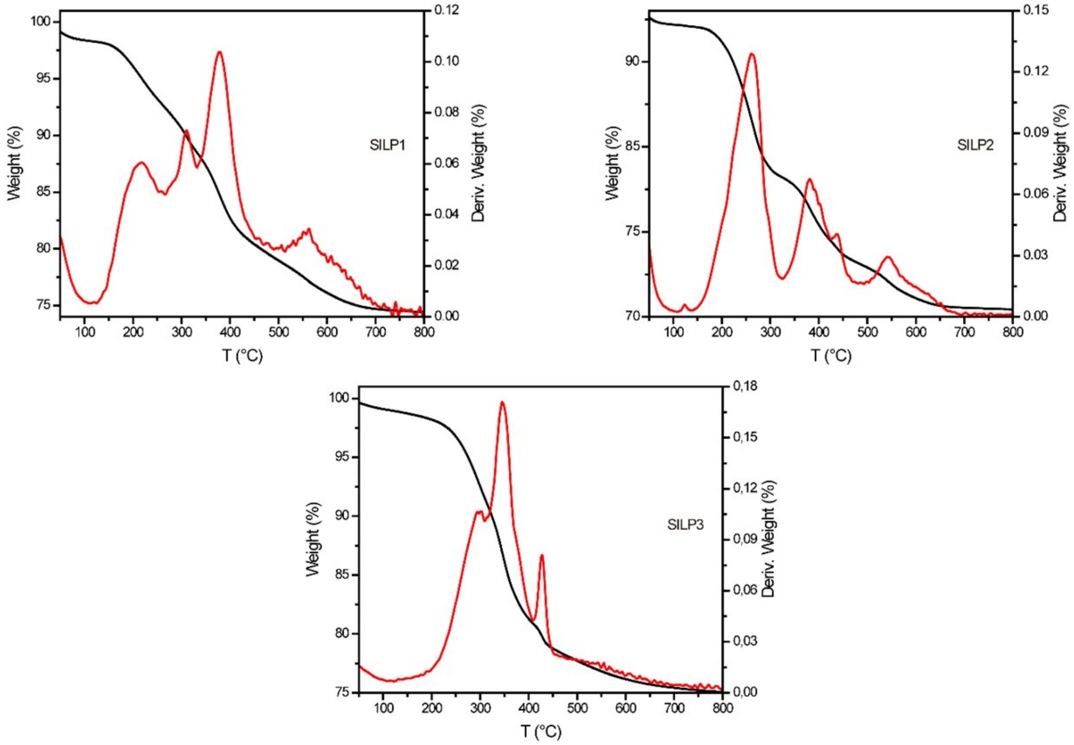

Figure S3. TGA corresponding to SILP1-3. In red DTG of SILP1-3.

1.4 X-rays Photoelectron Spectroscopy (XPS)

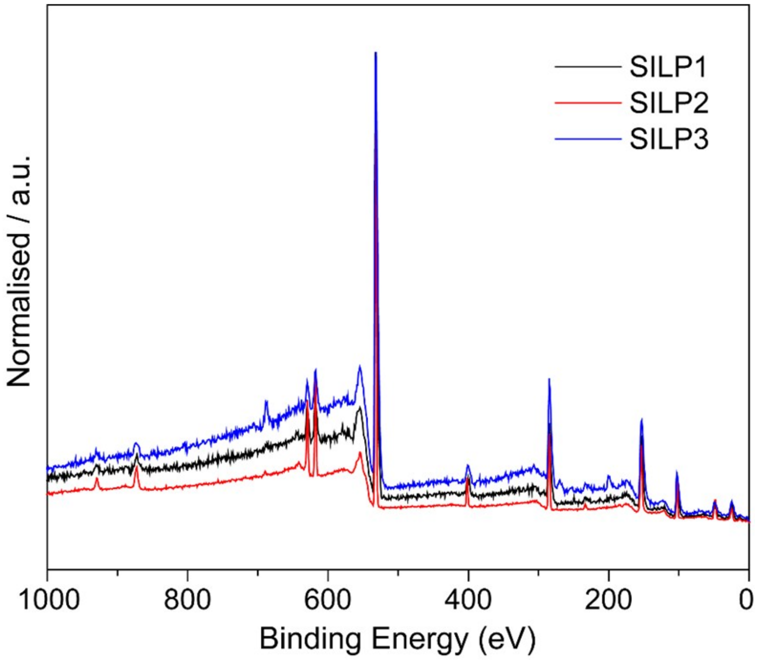

Figure S4. XPS at the long scan region of the SILP1-3 supports.

Table S3. Elemental composition of the surface of SILP1-3.

Entry	Sample	Cu 2p (%)	I 3d (%)	O 1s (%)	N 1s (%)	C 1s (%)	Si 2p (%)
1	SILP1		0.9	40.4	3.9	26.7	27.9
2	SILP2		1.3	41.1	4.9	27.0	25.4
3	SILP3		0.9	41.4	3.3	26.2	28.2
4	Cu@SILP1	2.6	0.5	37.8	5.4	32.0	21.5
5	Cu@SILP2	3.1	0.6	38.6	5.1	29.5	22.9
6	Cu@SILP3	2.8	0.4	36.2	5.8	35.4	19.7

1.5 Textural properties of SILPs

All the samples of two systems –SILP1-3 and Cu@SILP1-3 – were analysed through low-temperature nitrogen adsorption (T = 77.35 K, ASAP 2020, Micromeritics).

The pore size distribution (PSD) was estimated using three independent methods:

1) Semiempirical classical Barrett-Joyner-Halenda (BJH) approach based on Kelvin's equation predicting a pressure at which adsorbate will spontaneously condense (and evaporate) in cylindrical pores of a given size. The computation was carried out from the adsorption branch data.

2) Nguyen-Do method modified by Gun'ko (MND), which consists in the application of the self-consisted regularisation (SCR) under non-negative conditions of the pore size distribution function, i.e. $f(R_p) > 0$ at any pore radius R_p and the set regularisation parameter a = 0,01. For the present computation, the entire desorption branch was chosen. The method allows one to estimate the contributions of different pore types based on their size hierarchy: micropores (V_{micro} at $R_p < 1$ nm), mesopores (V_{meso} at 1 nm < R_p < 25 nm) and macropores (V_{macro}, $R_p > 25$ nm) in the distribution function $f_V(R_p)$. The pores were considered as cylinders and voids among particles aggregates.

3) Nonlocal density functional theory (NLDFT) in the model with the assumption for cylindrical pores using the adsorption branch. Unlike classical thermodynamic macroscopic models, the NLDFT methods describe the behaviour of fluids confined in the pores on a molecular level which allows molecular properties of gases to be related to their adsorption properties in pores of different sizes. It follows that pore size characterisation methods based on the NLDFT approach apply to the whole range of micro- and mesopores. The adsorbed fluid is to be on the tapis in a pore is in equilibrium with a bulk gas phase. It is a general practice to adjust interaction parameters (fluid-fluid and fluid-solid) in such a way that the model would correctly reproduce fluid bulk properties (*e.g.*, bulk liquid-gas equilibrium densities and

pressures, liquid-gas interfacial tensions) as well as standard adsorption isotherms on well-defined non-porous adsorbents. The calculation of pore size distribution is based on a solution of the Adsorption Integral Equation (AIE), which correlates the kernel of theoretical adsorption/desorption isotherms with the experimental sorption isotherm:

$$N\left(\frac{p}{p_0}\right) = \int_{W_{min}}^{W_{max}} N\left(\frac{p}{p_0},W\right) f(W)dW$$

where $N(p/p_0)$ - experimental adsorption isotherm data; W - pore width; $N(p/p0,W)$ - isotherm on a single pore of width W; $f(W)$ - pore size distribution function.

Specific surface area, S_{BET}, was calculated from the BET method. The total pore volume V_p was estimated at the maximal relative pressure of $p/p_0 \approx 0.98–0.99$ within the MND procedure in parallel to NLDFT and BJH.

The Fowler–Guggenheim (FG) equation was used to describe localised monolayer adsorption with lateral interaction:[1,2]

$$\theta_i(p,E) = \frac{Kp \cdot exp(\frac{zw\Theta}{k_B T})}{1 + Kp \cdot \exp(\frac{zw\Theta}{k_B T})}$$

where $K = K_0(T)exp(E/k_BT)$ is the Langmuir constant for adsorption on energetically uniform sites, and the pre-exponential factor $K_0(T)$ is expressed in terms of the partition functions for isolated gas and surface phases, z is the number of nearest neighbours of an adsorbate molecule (assuming $z = 4$), w is the interaction energy between a pair of nearest neighbours, k_B is the Boltzmann constant, e.g., $zw/k_B = 380$ K for nitrogen. This equation is used as a local isotherm, ϑ_1, in the overall adsorption isotherm equation, Θ, to calculate the distribution function $f(E)$ of the adsorption energy:

$$\Theta(T,p) = \int_0^{E_{max}} \theta_i(T,p,E) f(E)dE$$

where Θ is the experimental amount adsorbed. A maximum $p/p0$ value corresponds to nearly monolayer coverage $\Theta = a/a_m \sim 0.99$ [3]. This calculation was performed on the CONTIN-based software. [4,5]

The geometric topography of the surface structure of nanomaterials can be characterised by the fractal dimension D_S, which is a kind of roughness exponent. The determination of the surface roughness can be investigated using the modified Frenkel-Halsey-Hill (FHH) method, which is

applied in the range of multilayer adsorption.[6–8]

Hence, the estimation of nanoxide fractality was performed on the basis of the FHH method, which can be applied in the range of multilayer adsorption. For this purpose, the adsorption branch within $p/p_0 < 0.8$ values have been used, where the capillary condensation occurs in mesoporous SILPs. The fractal dimension D_S was being calculated from the plot corresponding to the following equation proposed by Avnir and Jaroniec:[9]

$$\ln(V/V_0) = (D_S - 3)\cdot\ln[\ln(p_0/p)] + \text{const}$$

where V, V_0 is the adsorbed volume of N_2, the saturation volume of adsorbed N_2, respectively, p and p_0 are the equilibrium and saturation pressure of nitrogen adsorbed, respectively.

Table S4. Textural characteristics of SILP1-3 and Cu@SILP1-3 calculated with MND method.

Sample	S_{BET} (m^2/g)	S_{nano}/S_{BET} (%)	S_{meso}/S_{BET} (%)	S_{macro}/S_{BET} (%)	V_p (cm^3/g)	V_{nano}/V_p (%)	V_{meso}/V_p (%)	V_{macro}/V_p (%)	R_p, (nm)
SILP1	130	0.0	99.4	0.6	0.236	0.0	90.9	9.1	10
SILP2	185	0.0	99.7	0.3	0.285	0.0	93.8	6.2	8
SILP3	306	0.0	99.6	0.4	0.405	0.0	92.7	7.3	8
Cu@SILP1	88	0.0	99.3	0.7	0.184	0.0	89.9	10.1	11
Cu@SILP2	220	0.0	99.7	0.3	0.315	0.0	94.6	5.4	7
Cu@SILP3	314	0.0	99.7	0.3	0.437	0.0	95.0	5.0	7

Table S5. Total pore volume, pore radius and specific surface area of pristine SILP1-3 and Cu@SILP1-3 calculated with BJH and NLDFT methods.

Textural properties	SILP1	SILP2	SILP3	Cu@SILP1	Cu@SILP2	Cu@SILP3
V_p, (cm^3/g)	0.226 (BJH)<br>0.221 (NLDFT)	0.258 (BJH)<br>0.270 (NLDFT)	0.336 (BJH)<br>0.381 (NLDFT)	0.183 (BJH)<br>0.172 (NLDFT)	0.279 (BJH)<br>0.300 (NLDFT)	0.339 (BJH)<br>0.387 (NLDFT)
R_p, nm	3 (BJH)<br>3 (NLDFT)	2 (BJH)<br>3 (NLDFT)	2 (BJH)<br>3 (NLDFT)	3 (BJH)<br>4 (NLDFT)	2 (BJH)<br>3 (NLDFT)	2 (BJH)<br>3 (NLDFT)
S_{BJH} (m^2/g)	124	153	201	94	171	208
S_{NLDFT} (m^2/g)	117	156	238	85	179	243

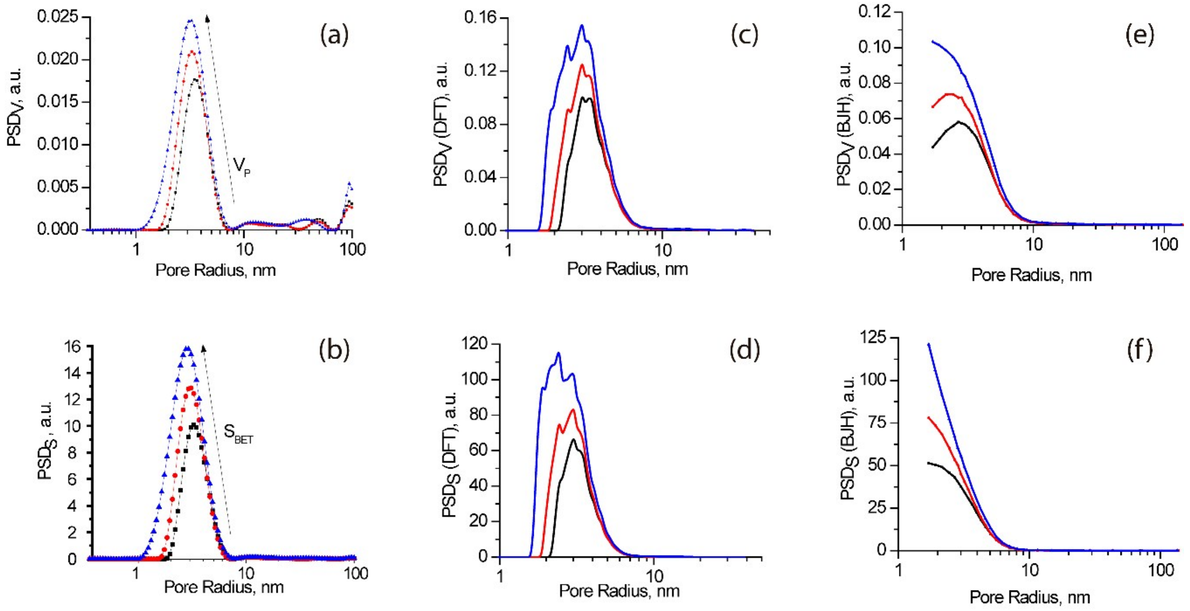

Figure S5. PSDs in volume (PSD_V) and surface (PSD_S) of SILP1-3 calculated through MND (a, b), NLDFT (c, d) and BJH (e, f) methods.

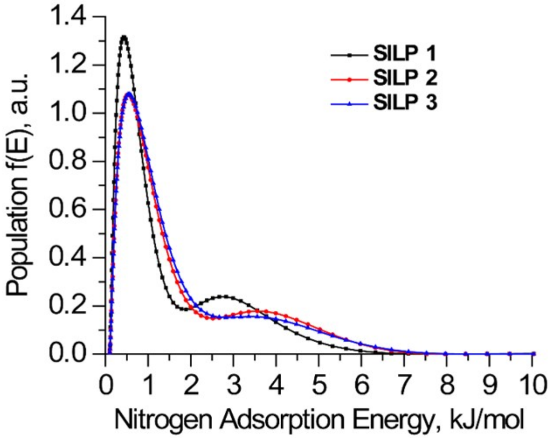

Figure S6. Nitrogen adsorption energy distributions for SILP1-3.

1.6 Field Emission Scanning Electron Microscope (FE-SEM)

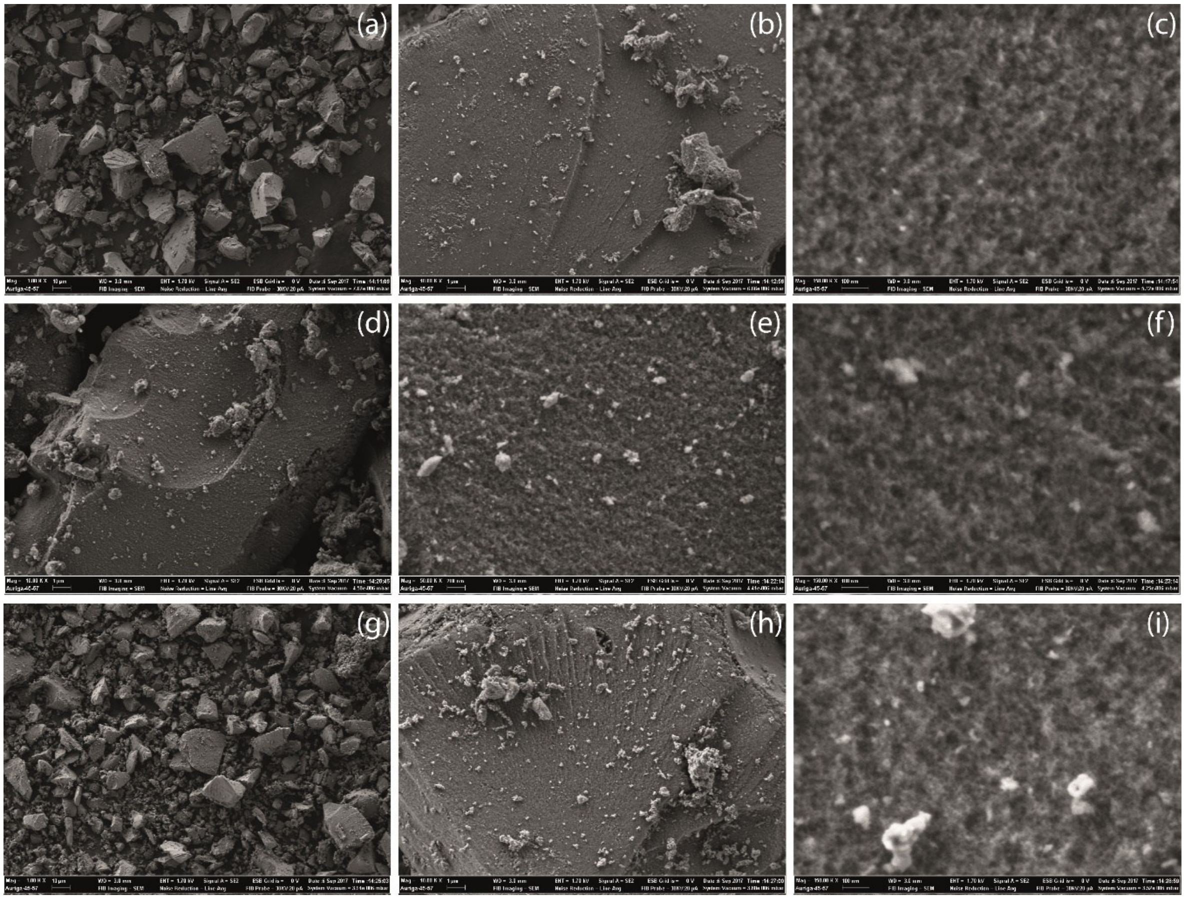

Figure S7. FE-SEM images corresponding to SILP1 (a, b ,c), SILP2, (d,e,f) and SILP3 (g,h,i).

2. Cu@SILPs Characterisation

2.1 Scanning Transmission Electron Microscope (STEM)

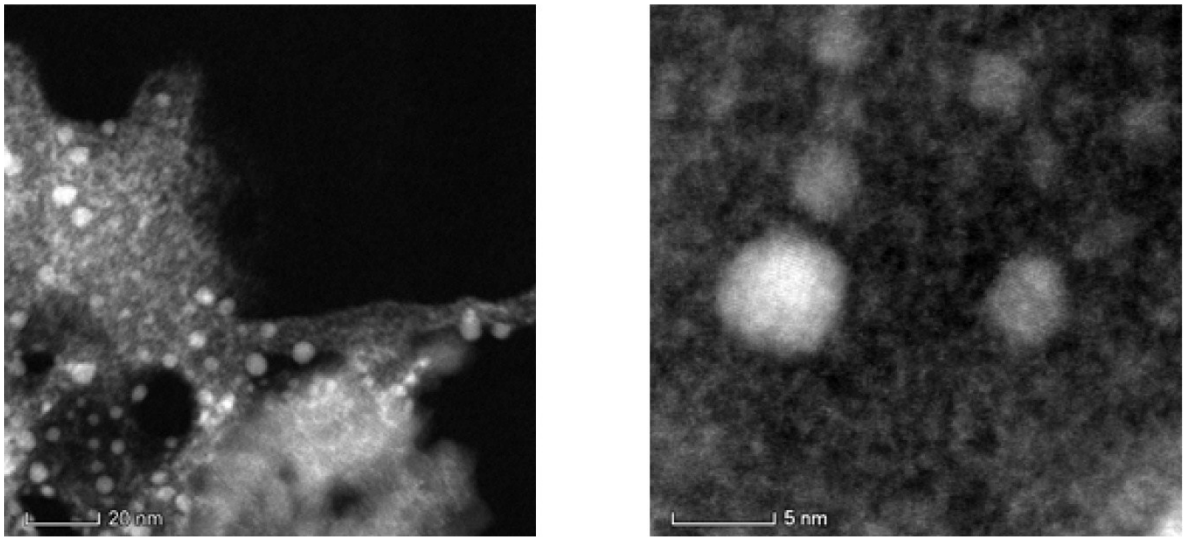

Figure S8. Selected STEM image of Cu@SILP1.

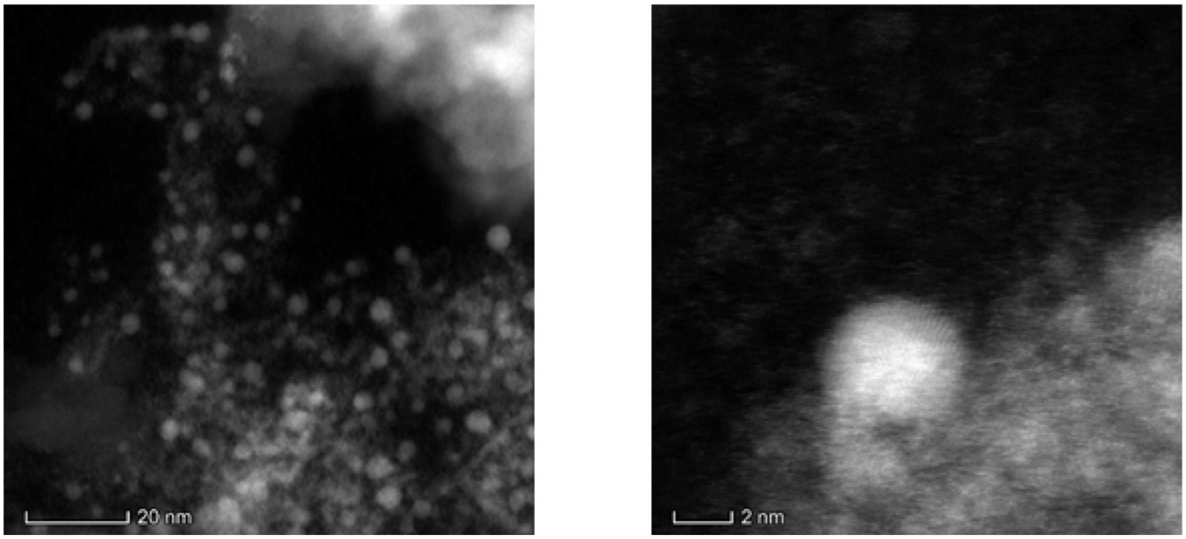

Figure S9. Selected STEM image of Cu@SILP2.

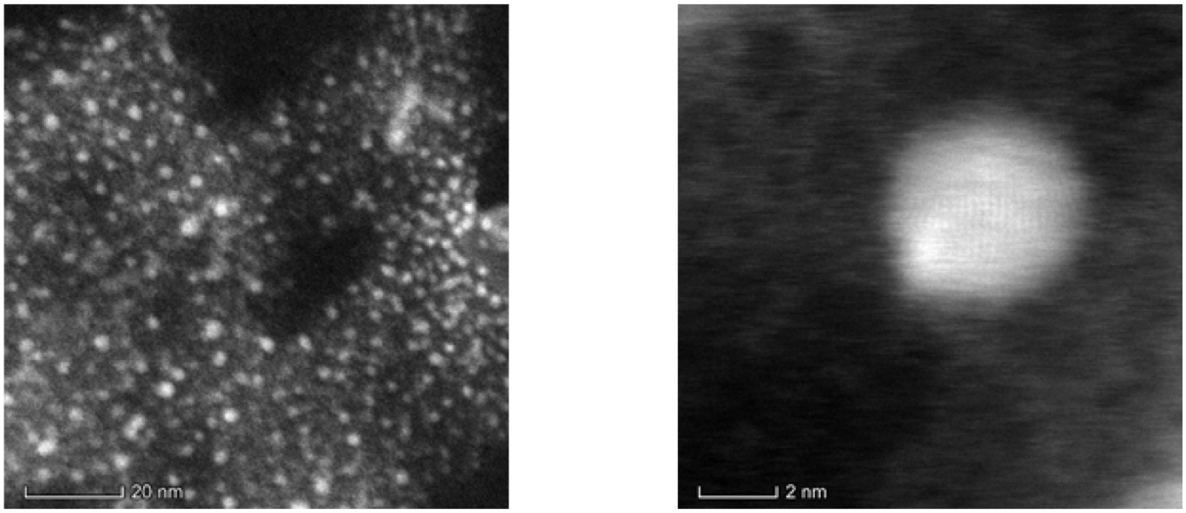

Figure S10. Selected STEM image of Cu@SILP3.

2.2 XPS analysis

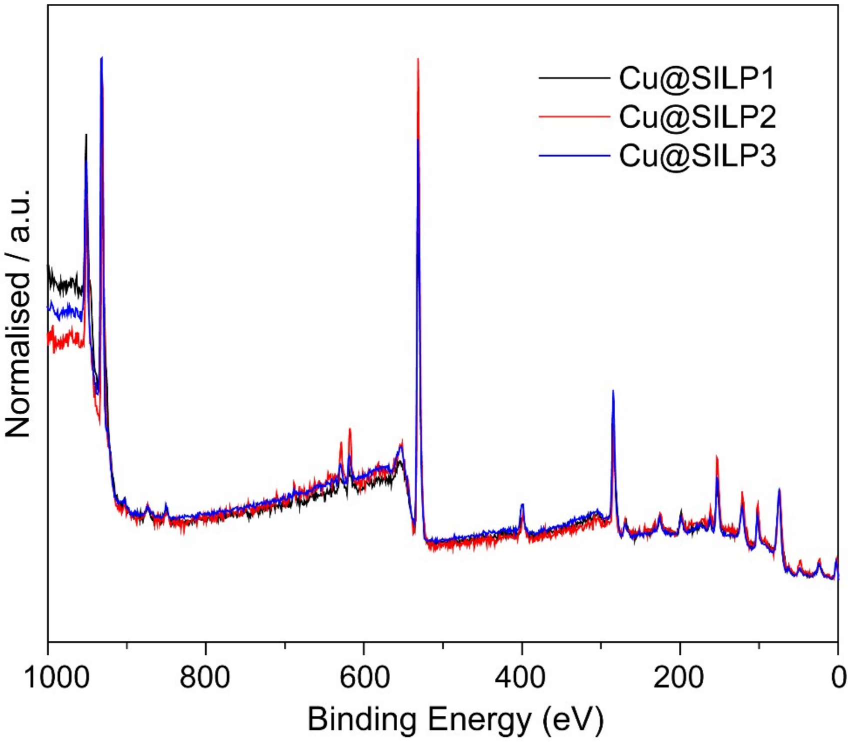

Figure S11. XPS at the long scan region of the Cu@SILP1-3 supports.

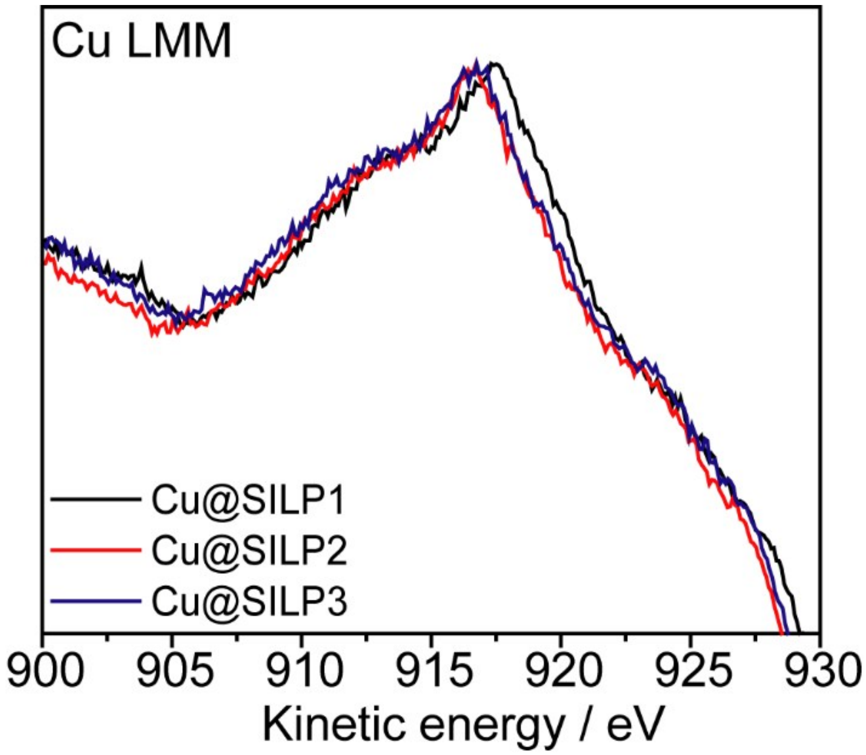

Figure S12. Cu LMM spectra of Cu@SILP1-3.

2.3 Textural properties

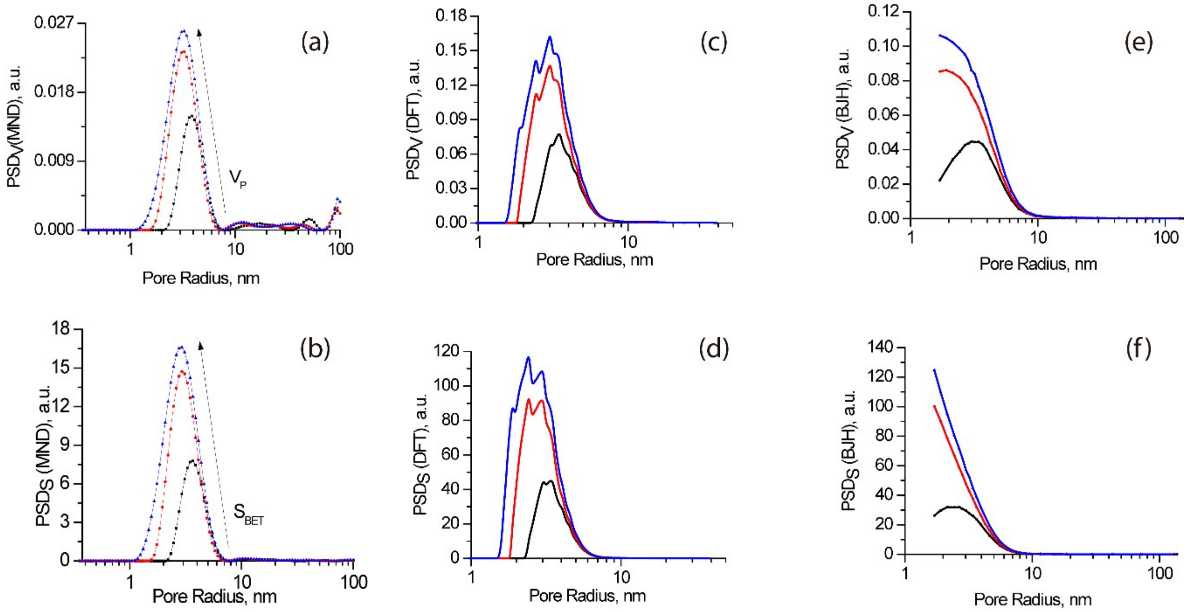

Figure S13. PSDs in volume (PSD_V) and surface (PSD_S) of for Cu@SILP 1-3 calculated through MND (a, b), NLDFT (c, d) and BJH (e, f) methods.

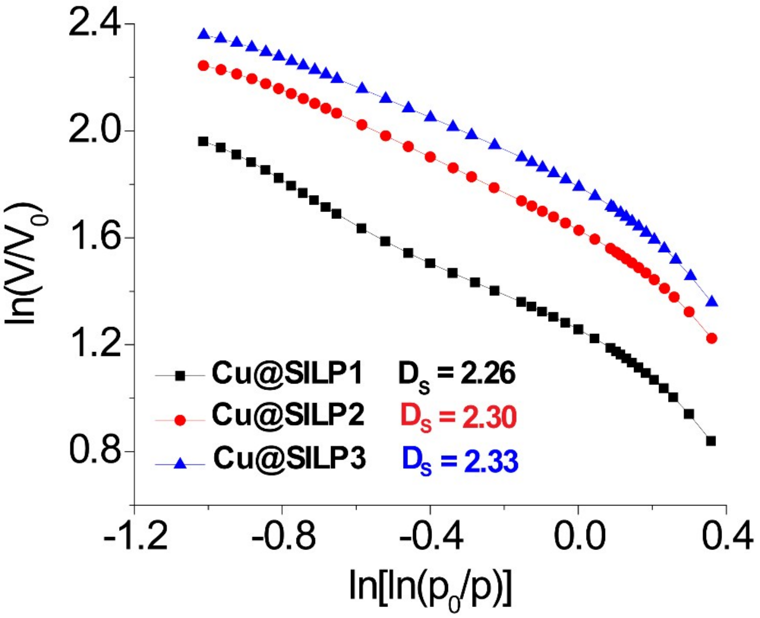

Figure S14. Frenkel-Halsey-Hill plot for Cu@SILP1-3.

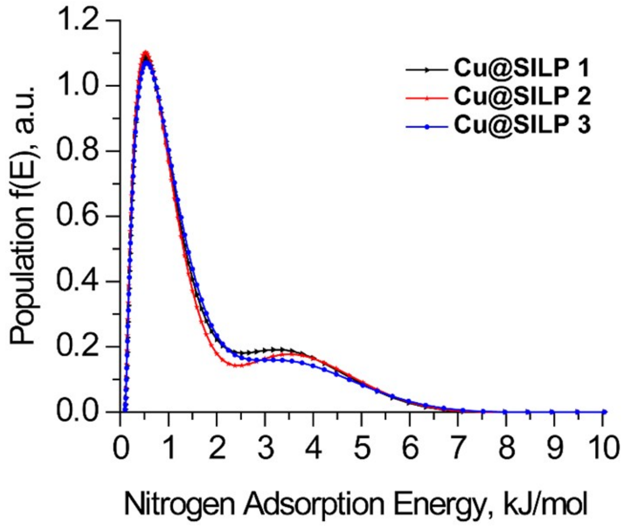

Figure S15. Nitrogen adsorption energy distributions for Cu@SILP1-3.

2.4 Catalytic Experiments

Table S6. Comparison of catalyst performance in the C-N Ullmann coupling between aniline and iodobenzene with different bases.

Entry	Cat	X	Base	Conv (%)	Sel (%)	TOF (h^1)
1	Cu@SILP 1	I	-	2	98	1
2	Cu@SILP 1	I	KOH	88	95	374
3	Cu@SILP 1	I	K_2CO_3	76	96	287
4	Cu@SILP 1	I	Cs_2CO_3	72	95	223
5	Cu@SILP 1	I	$N(Et)_3$	68	98	142
6	-[a]	I	KOH	0	0	0
7	Cu NPs[b]	I	KOH	60	90	149

Reaction conditions: Cu@SILP 1 (1 mol %), aniline (1.2 mmol), aryl halide (1 mmol), base (2 mmol), DMSO (3 mL), 100 °C, 4h. Conversion determined by GC. [a]Reaction without catalyst. [b]Purchased from ALDRICH

Table S7. Comparison of catalyst performance in the C-N Ullmann coupling between aniline and iodobenzene.

Entry	Cat	Base	Yield (%)	Reference
1	CuNPs@SILP1	KOH	88	This work
2	CuNPs@SiNWs	Cs_2CO_3	15	[10]
3	CuNPs/Zeolite	KOH	95	[11]
4	Cu_2O/graphene	NaOH	99	[12]
5	Cu/MnOx	K_2CO_3	5	[13]
6	$CuCl_3$-IL-SiO_2	K_2CO_3	95	[14]
7	CuCl/MWCNTs-Met	Et_3N	96	[15]

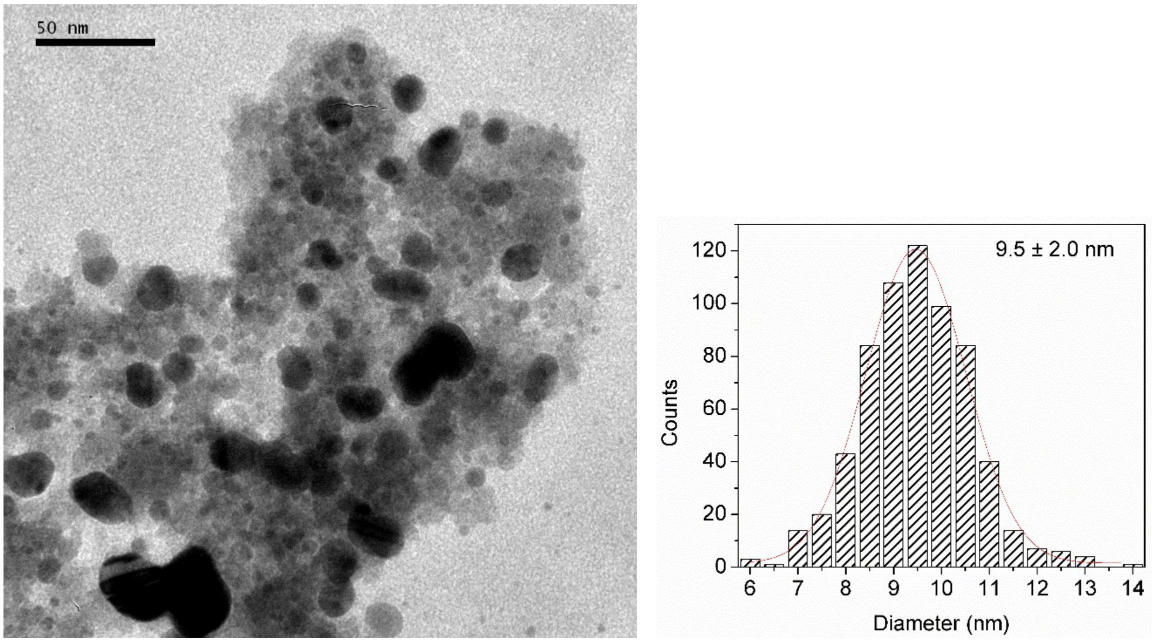

Figure S16. Selected TEM image of neat $Cu_2O@SiO_2$.

2.5 DFT method validation and Model Cluster

The computational method employed was selected according to a validation procedure based on empirical properties of the Cu_2 dimer[16] such as diatomic bond distance (*r*), Dissociation Energy (D_e), vibrational frequency (*f*), Vertical ionization potential (VIP) and the Electronic Affinity (EA). Calculations were performed with the relativistic pseudopotential and basis set LANL2DZ[17]. The functionals tested were PBE[18], BP86[19], OLYP[20] and M06L[21]. Table S9 summarizes the results obtained from the different functionals. The results indicate that lower average error is performed by the PBE functional with 6.02% of error with respect to the reference values. The worst performance was obtained from OLYP with a 13.73% of error, while, the meta-GGA functional M06L present an average error of ~10%. Considering these results, the functional selected to carry out the theoretical study is PBE.

Table S8. Summary of the results obtained from the calculation of r, D_e, f, VIP, EA, and their average error compared to experimental values extracted from[16].

Functional	r (Å)	D_e (eV)	*f* (cm^{-1})	VIP (eV)	EA (eV)	Error (%)
PBE	2.24	2.34	263	8.01	0.74	6.07

BP	2.23	2.36	267	8.14	0.92	6.62
OLYP	2.30	2.07	231	7.58	0.45	13.73
M06L	2.21	2.60	282	7.77	0.76	9.45
Exp.	2.22	2.01	264	7.90	0.83	-

3. References

1 C. P. Jaroniec, M. Jaroniec and M. Kruk, *J. Chromatogr. A*, 1998, 797, 93–102.

2 J. Choma and M. Jaroniec, *Langmuir*, 1997, 13, 1026–1030.

3 V. Gun'ko and T. J. Bandosz, *Phys. Chem. Chem. Phys.*, 2003, 5, 2096–2103.

4 S. W. Provencher, *Comput. Phys. Commun.*, 1982, 27, 213–227.

5 S. W. Provencher, *Comput. Phys. Commun.*, 1982, 27, 229–242.

6 M. Jaroniec, M. Kruk and J. Olivier, *Langmuir*, 1997, 13, 1031–1035.

7 A. B. Jarzębski, J. Lorenc and L. Pająk, *Langmuir*, 1997, 13, 1280–1285.

8 W. Xu, T. W. Zerda, H. Yang and M. Gerspacher, *Carbon N. Y.*, 1996, 34, 165–171.

9 D. Avnir and M. Jaroniec, *Langmuir*, 1989, 5, 1431–1433.

10 M. Casiello, R. Picca, C. Fusco, L. D'Accolti, A. Leonardi, M. Lo Faro, A. Irrera, S. Trusso, P. Cotugno, M. Sportelli, N. Cioffi and A. Nacci, *Nanomaterials*, 2018, 8, 78.

11 A. J. Raiza, K. Pandian and R. G. Kumar, *ChemistrySelect*, 2019, 4, 1964–1970.

12 Y. Wang, J. Wang, B. Wang, Y. Wang, G. Jin and X. Guo, *Catal. Surv. from Asia*, 2018, 22, 123–128.

13 K. Mullick, S. Biswas, C. Kim, R. Ramprasad, A. M. Angeles-Boza and S. L. Suib, *Inorg. Chem.*, 2017, 56, 10290–10297.

14 N. Yao and Y. Lin Hu, *Curr. Org. Chem.*, 2017, 21, 368–377.

15 E. Akhavan, S. Hemmati, M. Hekmati and H. Veisi, *New J. Chem.*, 2018, 42, 2782–2789.

16 D. Die, B.-X. Zheng, L.-Q. Zhao, Q.-W. Zhu and Z.-Q. Zhao, *Sci. Rep.*, 2016, 6, 31978.

17 P. J. Hay and W. R. Wadt, *J. Chem. Phys.*, 1985, 82, 299.

18 J. P. Perdew, K. Burke and Y. Wang, *Phys. Rev. B*, 1996, 54, 16533–16539.

19 A. D. Becke, *Phys. Rev. A*, 1988, 38, 3098–3100.

20 J. Baker and P. Pulay, *J. Comput. Chem.*, 2003, 24, 1184–1191.

21 Y. Zhao and D. G. Truhlar, *J. Chem. Phys.*, 2006, 125, 194101.
